# The herbal pharmacopoeia of Ecuador: a national model for integrating traditional knowledge and biodiscovery

**DOI:** 10.3389/fphar.2025.1662980

**Published:** 2025-10-30

**Authors:** Susana Llivisaca-Contreras, Jaime Naranjo-Morán, Martín Bastidas-Gálvez, Jairo Jaime-Carvajal, María Muenala-Tituaña, Patricia Manzano-Santana, Adrián Abad-Mihalache, Andrea Abril-Novillo, Juan M. Cevallos-Cevallos, Andrea Orellana-Manzano, Fabián León-Tamariz

**Affiliations:** ^1^ Centro de Investigaciones Biotecnológicas del Ecuador, CIBE, Faculty of Life Sciences, Escuela Superior Politécnica del Litoral, ESPOL, Campus Gustavo Galindo, Guayaquil, Ecuador; ^2^ Finca Botánica Aromática, Guayaquil, Ecuador; ^3^ Grupo de Investigación en Aplicaciones Biotecnológicas, GIAB, Carrera de Biotecnología, Universidad Politécnica Salesiana, UPS, Guayaquil, Ecuador; ^4^ Universidad Tecnológica ECOTEC, Guayaquil, Ecuador; ^5^ Fundación Kichwa Institute of Science Technology, KISTH, Language Department, Otavalo, Ecuador; ^6^ D. Gary Young Research Institute, Lehi, UT, United States; ^7^ Department of Biosciences, Group of Medicinal Plants and Natural Products, Faculty of Chemistry, Universidad de Cuenca, Cuenca, Ecuador; ^8^ Laboratorio de Farmacología Molecular Aplicada, Facultad de Ciencias de la Vida, Escuela Superior Politécnica del Litoral, ESPOL, Guayaquil, Ecuador

**Keywords:** medicinal plants, digital-ethnopharmacology, biodiversity, phytotherapeutics, scientific development, public health, complementary medicine

## Abstract

**Introduction:**

The Herbal Pharmacopoeia of Ecuador is a proposal aimed at systematizing and organizing information on the use of medicinal plants and natural products through the scientific evaluation of existing knowledge (ancestral or otherwise) in Ecuador, a country with high biodiversity and a rich ethnobotanical heritage, contributing to the safe use of medicinal plants and their potential phytopharmaceutical derivatives, offering safe alternatives for the treatment or relief of various health conditions.

**Objectives:**

To build a foundation that organizes and structures validated information on medicinal plants and natural products, serving in the future as a reference resource for the development of phytotherapeutic products, knowledge transfer, protection of ancestral knowledge, supported by regulatory bodies, and as a source of consultation for health professionals in Ecuador.

**Methodology:**

An interdisciplinary approach was used to develop monographs on medicinal plants used in various regions of Ecuador, integrating an extensive literature review that highlights ethnobotanical, pharmacological, and phytochemical analysis. In a collaborative effort by academic institutions integrated into the VLIR-Ecuador Network, a digital platform was developed using the Angular software framework to organize these monographs.

**Results:**

The creation of a digital platform enabled the systematization of scientific knowledge on 14 selected medicinal plants through the generation of monographs, organized within the Ecuadorian Herbal Pharmacopoeia. This has facilitated access for the medical and scientific community to relevant data on the common use of plants and traditional Ecuadorian medicine.

**Conclusion:**

The official adoption of an Herbal Pharmacopoeia in Ecuador will strengthen scientific production, support the regulation of natural products, protect ancestral knowledge, and promote research on bioactive compounds. Its success will depend on collaboration between the government, academia, industry, and ancestral communities, ensuring its development and positioning Ecuador as a leading reference in ethnobotany and biosustainability.

## 1 Introduction

Ecuador is considered one of the most biodiverse countries in the world, thanks to its diverse climate, geographical contrasts, and extensive biodiversity. The presence of the Andes Mountain range divides the country into three regions (Amazon, Highlands, and Coast), each with distinct cultural characteristics. Additionally, there is a fourth region: the Galápagos Islands. This extraordinary diversity is not only a source of national pride but also a reservoir of medicinal plant species, many of which have been used for centuries in traditional medicine (Malagón et al., 2016; Torre et al., 2006).

The use of plants for healing purposes is as old as civilization itself. In Ecuador, ancestral knowledge has been passed down from generation to generation, forming the foundation of the healthcare system for many Indigenous and rural communities (Moraes et al., 2007). However, despite this valuable ethnobotanical heritage, challenges remain in modern healthcare (Reinoso, 2008). Socioeconomic inequalities, limited access to medicines, and deficiencies in the public healthcare system have led many Ecuadorians to seek alternative treatments (SIT, 2020). As a result, medicinal plants remain an essential component of primary healthcare, especially in marginalized and rural populations (Moraes et al., 2007). Today, Ecuador risks losing its ancestral medicinal knowledge due to acculturation and the lack of an official record (Galindo Lozano, 2020; Malagón et al., 2016). This natural and cultural wealth not only offers the opportunity to develop effective therapeutic alternatives but can also strengthen the country’s healthcare system, in line with the World Health Organization (WHO) recommendations to promote practices based on traditional medicine (Burton et al., 2014).

Health problems around the world are diverse and complex, ranging from the growing issue of bacterial resistance and the emergence of variants of chronic diseases such as cancer, to the vulnerability of healthcare systems in the face of pandemics like COVID-19. Although antibiotics have been one of the most successful forms of chemotherapy, saving countless lives (Banin et al., 2017), the decline in the development of new drugs and the emergence of “superbugs” resistant to multiple medications (Alanis, 2005) have become major global health concerns (Mancuso et al., 2021). In Ecuador, hospital collapses and medicine shortages during the pandemic exposed the vulnerability of the healthcare system, highlighting the importance of exploring innovative alternatives such as the use of medicinal plants and ancestral knowledge, to strengthen the health response and address future crises sustainably (Llivisaca-Contreras et al., 2021).

In this context, the importance of a national pharmacopoeia becomes evident. The absence of an official herbal pharmacopoeia in Ecuador represents a significant gap in the regulating and validating medicinal plants and natural products (Mosquera, 2022). Many herbal remedies are marketed without scientific validation, and their therapeutic effects, active compounds, and potential toxicities remain largely undocumented. This not only poses a risk to consumers but also limits scientific and economic development in the field of phytotherapy (Dehesa, 2009). A well-structured compilation can close this gap by collecting validated information on medicinal plants, establishing quality standards, and promoting research. Creating an *Herbal Pharmacopoeia of Ecuador* would align the country with global efforts to integrate traditional medicine into modern healthcare systems. As is the case today, countries including China, India, or Germany have recognized the value of herbal medicine, incorporating it into their official health policies and developing comprehensive pharmacopoeias to standardize its use (Sharma, 2025). With its unmatched biodiversity, Ecuador has the opportunity to become a leader in this field. By systematically documenting medicinal plants, identifying bioactive compounds, and ensuring compliance with Good Agricultural Practices (GAP) and Good Manufacturing Practices (GMP), the country can establish a solid framework that benefits both health and the economy. Additionally, these efforts would help preserve ancestral knowledge, preventing its loss due to modernization and cultural changes (Durán et al., 2017; Mosquera, 2022; Ortega, 2025).

This paper outlines the initial efforts made through collaboration between various academic institutions and social organizations to lay the foundations for an Herbal Pharmacopoeia for Ecuador, compiling and organizing scientific information on various medicinal plants used in our country.

## 2 Methodology

### 2.1 Establishment of the FAPRONAT consortium for the herbal pharmacopoeia of Ecuador

To create and formalize the Ecuadorian Herbal Pharmacopoeia, it was necessary to establish the Consortium for the Pharmacopoeia of Plants and Natural Products of Ecuador (FAPRONAT), an initiative of the VLIR Network in Ecuador. The acronym VLIR (Vlaamse Interuniversitaire Raad) stands for the Flemish Interuniversity Council (VLIR-UOS) of Belgium (www.vliruos.be). Four Universities participated on the Ecuadorian site, Escuela Superior Politécnica del Litoral (ESPOL), Universidad de Cuenca (UCuenca), Escuela Politécnica Nacional (EPN), and Universidad Técnica del Norte (UTN). This network was active until 2024 (http://www.vlirnetworkecuador.com/) (https://webhistorico.epn.edu.ec/vlir-network-ecuador/).

FAPRONAT comprises scientists specializing in medicinal plants and natural products and is dedicated to pharmacological characterization and bioprospecting of secondary metabolites. It manages the Herbal Pharmacopoeia of Ecuador and is responsible for developing and validating monographs of the most in-demand native medicinal plants. Its goal is to disseminate the properties of the plants and promote their application in academia and industry, thereby contributing to the country’s scientific development.

### 2.2 Influence of pharmacopoeias on scientific output in health

A bibliometric analysis was conducted to understand the influence of pharmacopoeia on scientific output in the health field. Bibliometrics is a quantitative analysis method that uses mathematical and statistical tools to examine scientific production. It allows for predicting future research trends and summarizing large volumes of data and information (Regus et al., 2022). A comprehensive search of scientific publications was conducted in the Scopus database from 2000 to 2024. The year 2000 was chosen as the starting point, as it was when the genome of *Arabidopsis thaliana*, the first model plant whose genome (115.4 Mb) was sequenced (Kaul et al., 2000). *Arabidopsis thaliana* sequencing revealed essential genes for producing medicinal compounds, accelerating drug development. It was also a key genetic model for studying plant metabolic pathways, driving plant biotechnology and marking a turning point in botany and pharmacology (Tariq Yahya, 2022). The keywords for the bibliometric analysis were the following: scientific name, pharmacopoeia, herbal medicine, plant extract, toxicity, traditional use, and treatment. In this way, it is possible to demonstrate the scientific impact in a given country when an Herbal Pharmacopoeia is available and utilized to manage scientific and ancestral knowledge efficiently, influencing the health sector, phytotherapeutic production, and even the environmental sphere of a nation. The search equation is based on the methodology proposed by Manzano and Miranda (2015), who established three phases for the selection of journals: first, the identification of journals according to their topics; second, the analysis of titles, abstracts, and keywords based on relevance; and third, the selection of journals according to research quality criteria (Paoloni et al., 2023). This methodology allows for understanding the current state of pharmacopoeias research and identifying areas with the most significant potential for Ecuador and other Andean regions.

### 2.3 Design and construction of the monographs

A standard format was designed to develop the monographs of the Herbal Pharmacopoeia. Initially, these were developed based on the experience and scientific knowledge of the research groups regarding medicinal species, as previously mentioned. Likewise, internationally recognized monograph formats were analyzed, such as those of ESCOP (European Scientific Cooperative on Phytotherapy) based in the United Kingdom (Exeter), FEUM (Farmacopea de los Estados Unidos Mexicanos), the Brazilian Pharmacopoeia, the French Pharmacopoeia, and that of the WHO, etc. (CenadIM, 2025). We adapted specific ideas to our pharmacopoeia to align with the country’s interests. We established keywords and search criteria based on the proposed structure to gather information and draft the monographs. We consulted various digital databases, including ISI Web of Science, Scopus, PubMed, BioMed Central, Google Scholar, Tropicos.org, and The Plant List. To ensure that the Monograph is structured to organize existing scientific and traditional information effectively, it has been divided into three distinct Sections: 1) The first section provides information on identification and classification, focusing on its “safe use.” This includes scientific names, photographs, common names, botanical descriptions, micrographs, and genetic data. 2) The second section is dedicated to phytochemical composition and “pharmacological action.” It encompasses traditional uses, phytochemical profiles, pharmacological activities, toxicity or contraindications, extract preparation, and dosage guidelines. 3) The final section addresses “quality control,” compiling information on phytochemical and genetic markers, and rapid identification tests (Alamgir, 2017; WHO, 2019).

### 2.4 Monographs validation

To validate a complete monograph of a species, the double-blind method was used (Shmidt and Jacobson, 2022), establishing the following process: first, the editors send it to the consortium administrator; second, the administrator manages the peer review using the double-blind method; third, the reviewers evaluate the content and send their comments to the administrator, who in turn forwards them to the editors for the necessary corrections. This cycle is repeated until the monograph meets the scientific standards required for publication on the digital platform of the Herbal Pharmacopoeia of Ecuador.

### 2.5 Development of the digital platform for the herbal pharmacopoeia of Ecuador

The digital platform was initially built using the content management system “WordPress,” where different sections were created to structure the website. These included details such as the members of the FAPRONAT Consortium and their academic and scientific credentials, the services offered by the member universities, and scientific articles, among other options. Among these, the Monographs section was a key feature, designed as a repository for studies and research on various plants native to Ecuador. It covers topics ranging from their botanical descriptions and pharmacological activities to their toxicity-related contraindications, essential preparations, phytotherapeutic uses, and more.

This content management system, which remained in place until early 2024. From that year onwards, it was decided to enhance the platform’s technology to facilitate updates, improve cybersecurity, and adapt to the demands of a modern and globalized world. The redesign resulted in a new visual identity for the Pharmacopoeia on its website. The “Frontend” (the part the user interacts with) was developed using the “Angular” framework (Sahani, 2020), whose Single Page Application (SPA) functionality and use of components enable efficient design of the necessary sections. For the “Backend” (the functions that facilitate buttons, forms, and data submission), it is planned to be developed using the.NET Core framework, which will provide robust tools for managing data reception and database insertions, ensuring high levels of security against threats, scalability, and ease of maintenance (https://farmacopea.ec/). Social media accounts were also created for the *Herbal Pharmacopoeia of Ecuador,* where monographs and events such as courses, workshops, seminars, webinars, and conferences will be shared.

**FIGURE 1:**
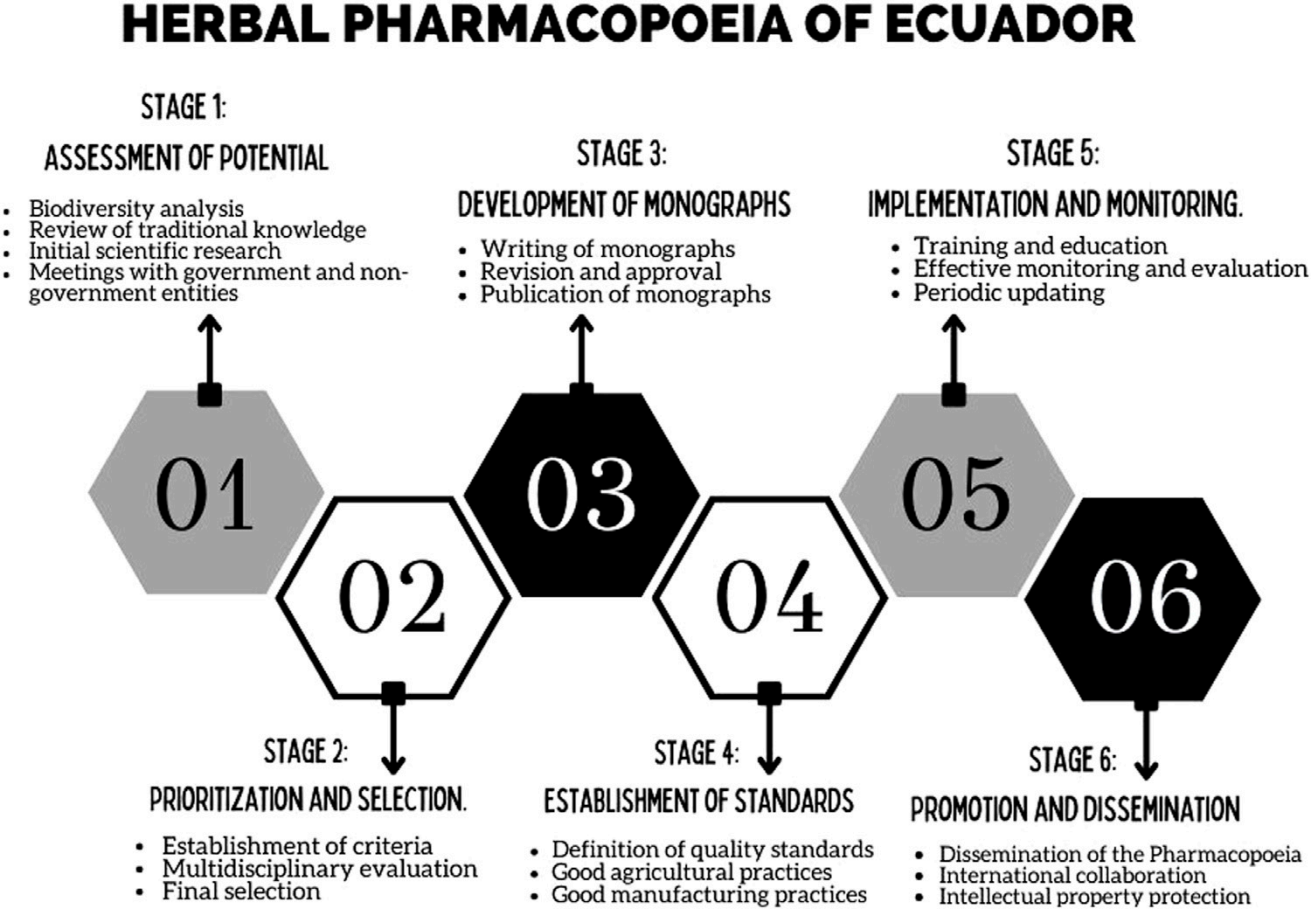
Structuring of the official Pharmacopoeia of Ecuador, coordinated by the VLIR Network Ecuador in the year 2020.

The structure of the official Pharmacopoeia was developed in several stages, as shown in Figure 1. To date, we have completed Stage 5 and are currently in Stage 6, corresponding to “promotion and dissemination.” It is worth emphasizing that the duration of each stage depends on the funding obtained and the current government’s priorities for supporting the project. We will continue to seek resources, with the expectation that, in the future, the Herbal Pharmacopoeia of Ecuador may be incorporated into the annual state budget.

### 2.6 Engagement with regulatory entities and official instruments

A participatory and regulatory engagement framework was implemented in the research design to ensure institutional alignment, legal compliance, and cultural inclusivity for the development of the Herbal Pharmacopoeia and the FAPRONAT initiative. The methodology involved formal consultations, collaborative agreements, and multi-sectoral dialogues with key national agencies responsible for intellectual property, environmental protection, and health regulation. Initially, coordinated meetings were held with the Servicio Nacional de Derechos Intelectuales (SENADI) to address the protection of Indigenous intellectual property rights. As a result of these discussions, a cooperative agreement was established with the Kichwa Institute of Science, Technology and Humanities (KISTH). This agreement enabled a systematic review and bilingual translation of selected monographs into the Kichwa language, ensuring linguistic accessibility and cultural validation of traditional knowledge. Next, the project team collaborated with the Ministry of the Environment, Water, and Ecological Transition (MAATE, acronym in Spanish) to assess the pharmacopoeia’s contributions to biodiversity conservation and sustainable resource use. MAATE provided institutional support, affirming that the initiative aligns with national conservation goals, particularly concerning endemic medicinal species. Finally, the Agencia Nacional de Regulación, Control y Vigilancia Sanitaria (ARCSA) proposed technical collaboration to integrate the Herbal Pharmacopoeia into national pharmacovigilance and natural product monitoring frameworks. These collaborative efforts were structured through inter-institutional working sessions, during which the Pharmacopoeia’s scientific and regulatory utility was assessed. All institutional interactions followed a structured protocol that involved preparing briefing documents, presenting scientific justifications, and integrating feedback into the project design. Emphasis was placed on aligning the Pharmacopoeia with Article 385 of the Ecuadorian Constitution, which mandates support for scientific innovation, protection of traditional knowledge, and sustainable development. Efforts are ongoing to secure official recognition of the Herbal Pharmacopoeia and FAPRONAT through formal endorsement processes. This step is crucial for establishing regulatory legitimacy, fostering scientific collaboration, and ensuring equitable access to and responsible use of ancestral medicinal knowledge.

## 3 Results

### 3.1 Influence of pharmacopoeias on scientific production worldwide

**FIGURE 2:**
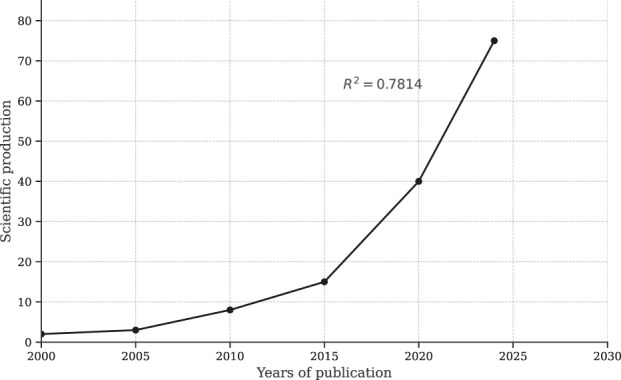
Positive trend in scientific production from 1995 to 2024, based on data obtained in March from the Scopus repository.

**FIGURE 3:**
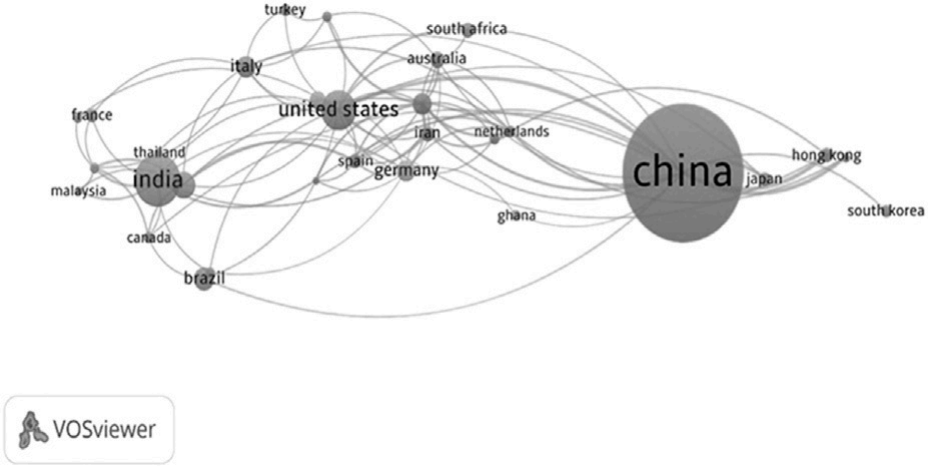
VOSviewer overlay visualization shows the scientific production of different countries related to pharmacopoeias and natural products from 2000 to 2024. The network map shows the interconnection of the main pharmacopoeias, with the size of the nodes representing the relevance and normative production of each country. China, India, the United States, Germany, and Brazil are highlighted as central players. The connections reflect collaboration and alignment between national, regional, and international pharmacopoeias, as described in the WHO Index of Pharmacopoeias (WHO, Working document QAS/11.453/ Rev.17). This network illustrates the importance of global harmonization of standards for medicine quality, safety, and efficacy, within the framework of initiatives like the European Pharmacopoeia and the WHO’s coordinated work.

The search results for “Pharmacopoeia” and “natural products” yielded a total of 576 scientific documents published between 2000 and 2024 on the Scopus platform. As depicted in Figures 2, 3, a noticeable upward trend is evident in scientific publications, indicating that ongoing research is progressively bridging existing gaps in the field. It is anticipated that the number of publications will continue to grow within the international scientific community in the years ahead.

By utilizing the VOSviewer tool, we assessed countries with official pharmacopoeias based on their bibliometric output and arranged them in descending order. The seven leading countries are China, India, the United States, Morocco, Brazil, Germany, and Italy, with publication totals of 231, 63, 43, 25, 21, 19, and 19, respectively. In the Americas, the United States and Brazil emerge as the most significant contributors (see Figure 3).

The United States Pharmacopoeia (USP) holds significant global influence and adheres to rigorous quality standards, making it a prevalent choice within the pharmaceutical industry. In contrast, the Brazilian Pharmacopoeia is primarily focused on traditional medicine and specifically caters to the needs of its local population. Other major pharmacopoeias, such as the Japanese Pharmacopoeia (JP) and the European Pharmacopoeia (Ph. Eur.), are in the process of harmonizing their General Chapters to promote consistency. Additionally, the World Health Organization (WHO) has published a series of monographs on selected medicinal plants, which includes five volumes—four initial volumes plus an additional one dedicated to newly independent states. While these monographs are not legally binding, they provide a valuable global reference, offering comprehensive and scientifically rigorous information on medicinal plants.

Pharmacopoeias showcase cultural diversity and various medical traditions; however, they all share a common focus on ensuring the quality and safety of medicines. In our increasingly interconnected world, harmonizing these standards is essential for facilitating international trade and enhancing access to safe and effective treatments. According to the Ministry of Public Health of Ecuador (Art. 26), the following pharmacopoeias are recognized as normative codes: the United States Pharmacopoeia, the National Formulary of the United States, the British Pharmacopoeia, the International Pharmacopoeia, the European Pharmacopoeia, the French Codex, and the Chinese Pharmacopoeia. These serve as references for processed natural products intended for medicinal use (Reglamento de Registro Sanitario Para Medicamentos En General, 2021). This selection, along with other globally significant codes, supports the analysis presented in Table 1.

**TABLE 1:** Comparison of selected international reference pharmacopoeias (https://www.who.int/publications/m/item/QAS-11.453-Rev.12).

Pharmacopoeias	European scientific cooperative on Phytotherapy-ESCOP	Japanese pharmacopoeia	European pharmacopoeia	USP (United States)	Korean pharmacopoeia	Mexican pharmacopoeia	Brazilian pharmacopoeia	French codex (pharmacopoeia)	Indian pharmacopoeia ayurveda	Chinese Pharmacopoeia-MTC)	WHO pharmacopoeia
Main Objective	Promote the safe and effective use of phytotherapeutics	Establish quality standards for medicines and some natural product	Establish quality standards for medicines	Establish quality standards for medicines	Establish quality standards for medicines and traditional products	Establish quality standards for Mexican medicines and natural products	Establish quality standards for medicines and phytotherapeutics	Provide national reference standards for medicines and herbal products	Standardize quality, safety, and efficacy of Ayurvedic and traditional medicines	Standardize quality, safety, and efficacy of traditional Chinese medicines	Provide international standards for the quality, safety, and efficacy of medicines and herbal products
Focus	Phytotherapy	Medicines and natural products	Medicines in general	Medicines, biological products, and medical devices	Traditional Korean medicine and conventional medicines	Conventional and traditional Mexican medicine, including herbal products	Phytotherapy and conventional medicines	Medicines, herbal drugs, and preparations	Ayurvedic medicine and products: herbs, minerals and natural compounds	Traditional Chinese medicine: herbs, formulas, minerals, and animal products	Essential medicines, herbal and traditional products
Content	Detailed monographs on medicinal plants and their preparations	Monographs on substances, pharmaceutical products, and some natural products	Quality, purity, and testing requirements for medicines	Detailed monographs, tests, and procedures to ensure quality	Monographs on traditional Korean products and conventional medicines	Monographs on Mexican natural products and conventional medicines	Monographs on herbal medicines and conventional medicines	Detailed monographs on substances and pharmaceutical products	Monographs on herbs, minerals, Ayurvedic preparations, and natural products; quality tests	Monographs on herbs, herbal formulas, minerals, and animal medicines; preparation methods and quality tests	Monographs on pharmaceutical substances, herbal medicines and guidelines for good practices
Influences	Traditional European medicine, especially German	Traditional Chinese, Japanese medicine plus Western standards	European pharmacopoeias and its own pharmaceutical tradition	Collaboration of EU member states and influenced by USP and WHO	Traditional Korean medicine combined with Western pharmaceutical science	Traditional Mexican medicine combined with international standards	Brazilian traditional medicine and indigenous knowledge, combined with European standards	Long French tradition in pharmacy, herbalism, and European standards	Ayurveda, classical texts (Charaka, Sushruta), traditional Indian practice	Traditional Chinese medicine, historical texts, clinical experience, and modern pharmacology	International collaboration, evidence-based medicine, traditional knowledge from multiple regions
Legislation	Influences the legislation of many European countries on phytotherapy	Legal basis for pharmaceutical regulation in Japan	Legal basis for pharmaceutical regulation in many European countries	Legal basis for pharmaceutical regulation in the U.S.	Legal basis for pharmaceutical regulation in South Korea	Legal basis for pharmaceutical regulation in Mexico	Legal basis for pharmaceutical regulation in Brazil	Legal basis for pharmaceutical regulation in France	Legal standard for regulation of Ayurvedic and traditional medicines in India	Legal standard for registration, production, and quality control of TCM in China	Reference for national regulatory authorities to harmonize pharmaceutical standards
Organization	ESCOP, a scientific cooperative	National Pharmaceuticals Agency of Japan	European Directorate for the Quality of Medicines (EDQM)	United States Pharmacopoeial Convention (USP)	Ministry of Food and Drug Safety of South Korea	Comisión Federal de Protección contra Riesgos Sanitarios-COFEPRIS	Agencia Nacional de Vigilancia Sanitaria (ANVISA)	Agence nationale de sécurité du médicament et des produits de santé (ANSM)	Indian Pharmacopoeia Commission, Ministry of Health & Family Welfare	Chinese Pharmacopoeia Commission, National Medical Products Administration (NMPA)	WHO, Expert Committees and collaborating centers worldwide

**FIGURE 4:**
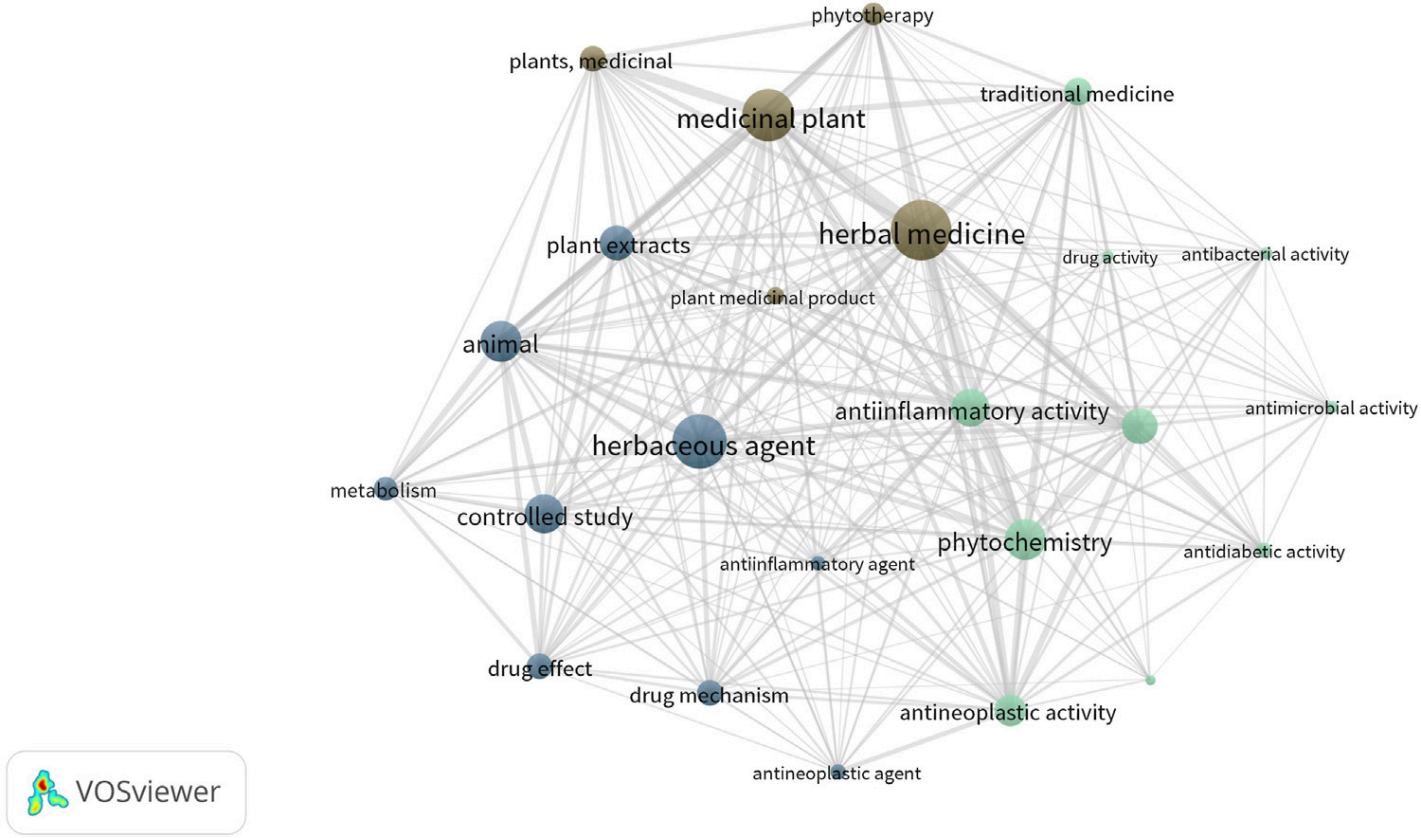
Network visualization of terms related to herbal medicine and its biological activities. In the network visualization generated by VOSviewer, the size of each node (dot) reflects the frequency of occurrence of the corresponding term in the analyzed literature; larger nodes indicate greater frequency and relevance within the dataset. The colors represent groupings of frequently co-occurring terms, according to the software’s clustering algorithm. Each color corresponds to a distinct thematic cluster, allowing the reader to visually identify conceptual relationships and research trends in the field of medicinal plants and their pharmacological activities.

Through this bibliometric analysis, several significant thematic areas were identified. The most notable among these include pharmacology, toxicology, and pharmacy, which together accounted for 367 documents; medicine, with 194 papers; and biochemistry, genetics, and molecular biology, which comprised 136 relevant scientific works compiled over the past 24 years. The six most prevalent keywords identified in the scientific articles were “phytochemistry,” “therapeutics,” “chemistry,” “articles,” “herbal medicine,” and “medicinal plants,” as depicted in Figure 4. Utilizing these keywords for information searches and text drafting revealed “herbal medicine” as a central theme that both connects and flourishes in areas where research gaps exist. This also highlights opportunities for development and innovation in the fields of medicines and medicinal plants (refer to Figure 4).

Brazil is at the forefront of scientific production in this field, with Argentina and Peru closely behind. Argentina is especially recognized for its research in traditional medicine and phytotherapy. Other countries demonstrating notable scientific contributions include Bolivia, Colombia, Chile, and Uruguay, while Ecuador ranks the lowest in scientific output (Table 2).

**TABLE 2:** Scientific production of the leading South American countries on pharmacopoeia and natural products between 2000 and 2024.

Range	Country	Documents	Range	Country	Documents
1	Brazil	21	5	Chile	3
2	Argentina	8	6	Colombia	3
3	Perú	4	7	Uruguay	3
4	Bolivia	3	8	Ecuador	2

### 3.2 Herbal pharmacopoeia of Ecuador

The Herbal Pharmacopoeia of Ecuador was established in response to the health crisis triggered by the COVID-19 pandemic in 2020. During this period, shortages of pharmaceutical products led to a heightened reliance on medicinal plants, often which, due to lack of guidance, posed significant health risks. In light of this situation, the need to create a reliable digital platform about the use of medicinal plant and natural products (https://farmacopea.ec) was recognized.

This platform currently features 14 monographs on selected different plant species, selected for their widespread traditional use and relevance to Ecuadorian traditional medicine. They represent a diverse range of botanical families and life forms, from trees such as *Theobroma cacao* to herbs such as *Artemisia annua*. The selection encompasses species native to the Andean and Amazonian regions, such as *Ilex guayusa*, along with introduced and cultivated plants such as *Azadirachta indica*, reflecting a mix of local and global flora. Considering, furthermore, the limited organization of research on these organisms in our country, the species were selected based on the knowledge generated about them by the different research groups at the FAPRONAT membersAs described, 14 medicinal plants were selected for the development of the monographs, namely: *Azadiractha indica* A. Juss., *Conyza bonariensis* (L.) Cronquist, *Ilex Guayusa* Loes., *Vernonanthura patens* (Kunth) H. Rob., *Mansoa alliacea* (Lam.arck) A.H.Gentry, *Passiflora edulis* f. flavicarpa O. Deg., *Theobroma cacao* L., *Vallesia glabra* var. glabra (Cav.) Link, *Artemisia annua* L., *Clinopodium nubigenum* (Kunth) Kuntze, *Jungia rugosa* Less., *Minthostachys mollis* (Kunth) Griseb., *Peperomia galioides* Kunth in H. B. K., and *Carica pentagona* Heilborn. Their main reported activity is presented in Table 3. Verification of taxonomy, scientific names, and/or synonyms was carried out using the botanical databases of the Field Museum (plantidtools.fieldmuseum.org), the Missouri Botanical Garden (mobot.org), and Tropicos (https://www.tropicos.org), which are reliable sources for the taxonomy of plants in the Neotropical region and Ecuador.

**TABLE 3:** Selected medicinal plants for the development of monographs, including their main pharmacological activities, traditional uses, key bioactive compounds, and the validation methods reported in the scientific literature.

Specie	Family	Common names/Spanish and/or kichwa	Plant´s structure	Pharmacological activities	Identified compounds	Traditional use	References	Validation
*Azadiracthta indica* A. Juss	Meliaceae	Castilian: Neem, nim, margosa, lila india, nimmi, limba, limbo, nimba, imba, mambo	Branches without bark; leaves and seeds	Antidiabetic, Antioxidant, Anticancer, Antiviral, Antimicrobial, Antifungal, Anti-inflammatory	Alkaloids, anthroquinones, terpenoids (nimbinene, nimbinal, nimbandiol, salanine, nimbidiol, ferruginol), sterols, saponins, flavonoids and tannins	Skin ulcers, hair loss, constipation, insecticide, intestinal worms, phlegm and washing wounds	Agrawal et al. (2020), Kale et al. (2020), Singh et al. (2020), Yarmohammadi et al. (2021)	*In vitro* and *in vivo* anticancer, anti-inflammatory
*Conyza bonariensis* (L.) Cronquist	Asteraceae	Castilian: Canilla de venado, Rama negra, Pata de venado, Mata negra o Hierba carnicera Kichwa: Aya Watchi	Whole plant	Antioxidants; Antibacterial; Antimycotic; Hepatoprotective; Anticancer; Anti-inflammatory; Antioxidant Activity	Essential oil: trans-β-farnesene, caryophyllene oxide, caryophyllene, and globulol Biochemical level: phenolic acids, flavonoids, terpenoids, tannins, and saponins	Skin disorders, fungal and bacterial infections, wounds, constipation, diarrhea, headache, rheumatism, cystitis, nephritis, leucorrhea, gastroenteritis, and menorrhagia	de Paula et al. (2018), Manzano et al. (2011), Saleem et al. (2014), 2015; Thabit et al. (2015)	*In vivo* antibacterial effect in rats; hepatoprotective; Activities antibacterial and antimicotic *in vitro*
*Ilex Guayusa* Loes	Aquifoliaceae	Castilian: Guayusa, Agracejo, Citrodora Kichwa: Guayusata, Wayusa panka, Wayusa, Waisa	Leaves	Anti-inflammatory activity, Antidepressive, Estrogenic, Estimulant	Caffeine (leaves), theobromine, theophylline, chlorogenic acids, ursolic acid, catechins and epicatechins, L-theanine, quercetin, carotenoids (α- and β-carotene, lutein), tannins and vitamin C	Antidiarrheal, tonic in steam baths and to relieve colic, fever, pain, rheumatism, depression and snake bites and as a fertilizer	Ochoa-Ocampo et al. (2025), Radice et al. (2017)	*In vivo* estrogen agonist
*Vernonanthura patens* (Kunth) H. Rob	Asteraceae	Castilian: Laritaco, Chilca, Varejón, Pebetero, Cusuco, Tuete, Sukunang, Xuqunán, Sanalego Kichwa: Añangu butsi yura	Leaves, flowers and branches	Antioxidant; Antiparasitic; Antimicrobial; Antifungal; Antifungal, Citotoxicity Activity	Lupeol and epilupeol, triterpenes and steroids, catechins, lactones, phenols, tannins and saponins	Dysentery, headache, fever, snakebite, athlete’s foot, stomachache, skin rashes, malaria, labor and postpartum pains, diarrhea, anthelmintic, cough	Isabel et al. (2012), Manzano Santana et al. (2012), Manzano and Miranda (2015)	*In vitro* antioxidants; antiparasitic; citotoxicity
*Mansoa alliacea* (Lam.) A. H. Gentry	Bignoniaceae	Castilian: Ajo de monte, Sacha ajo, Cipo-alho, Alho– damata, Bejuco de ajo Kichwa: Wiyagen	Root, stem, leaves and flowers	Antioxidant; Anticancer; Antimicrobial; Antifungal; Antifungal; Antiparasitic; Larvicidal; Antimalarial; Vermifuge; Antimicrobial activity	Alkaloids, tannins, phenols, saponins, flavonoids and quinones	Headache, abdominal pain, rheumatic and muscular pain, fever, cough, purgative, fatigue, anemia, nausea, constipation, infections, colds, blood pressure and repellent	Condori et al. (2013), Hamann et al. (2019), Rao et al. (2019), Salazar et al. (2017), Sudirga et al. (2019)	*In vitro* cytotoxicity against tumor cell lines
*Passiflora edulis* f. flavicarpa O. Deg	Passifloraceae	Castilian: Maracuyá, Parchita, Fruta de la pasión, Grenadelle, Granadina, Pasiflora Kichwa: Tintín, Apincoya	Leaves, fruit, pulp, seed and peel	Antioxidant; Antibacterial; Antifungal; Hepatoprotective; Anti-inflammatory; Antidiabetic; Antitumor; Anticancer; Analgesic; Antihypertensive and Cardioprotective Activity	Vitamin C, carotenoids, anthocyanins, flavonoids; Linoleic acid, oleic acid, palmitic acid; β-sitosterol, stigmasterol, squalene; Ionol, palmitaldehyde, ethyl palmitate, linalool; Amino acids	Dietary consumption. For hypertension, diarrhea, infant colic, menopausal symptoms, sedative, digestive, tonic, diuretic, anthelmintic	He et al. (2020), Pereira et al. (2023), Rai et al. (2022), Zhang et al. (2023)	*In vivo* anti-inflammatory effect gastrointestinal tract; antihypertensive
*Theobroma cacao* L	Malvaceae	Castilian: Cacao, Cacao arisco, Cacao común, Cacao criollo, Cacao dulce, Cacao silvestre Kichwa: Cacahua caspi	Bark: Inflamed kidneys, dysentery, cough, scabies Leaves: Headache, toothache, and diarrhea	Cardiovascular; Antioxidant; Neuronal; Antiobesity; Antidiabetic; Antiviral; Cosmeceutical potential; Cardiovascular Activity	Fatty acids, amino acids, alkaloids such as methylxanthines and purines, sterols, aromatic compounds, vitamins, phenols, flavonoids and terpenes	Peel and leaves	Giacometti et al. (2016), Jean-Marie et al. (2022), Sitarek et al. (2024), Żyżelewicz et al. (2016)	Antioxidant, anti-inflammatory, anticancer and antinecrotic properties *in vivo* and PTP1B inhibition and cytoprotective activity *in vitro* against oxidative stress in mice
*Vallesia glabra* var. *glabra* (Cav.) Link	Apocynaceae	Castilian: Cuncuno, Cun Cun, Perlilla, Perlillo, Peralillo, Huevito, Cacarahua, Ancoche	Leaves: for snake bites, diarrhea, blood vomiting, skin conditions, fever, cough, sore throat, and earache. The branches are used as an infusion for ulcers. Root: for measles, rheumatism, and muscle pain	Neuroprotective; Anti-inflammatory; Anticancer; Antiviral; Antifungal; Germicidal; Antitussive; Antitussive	Terpenes, saponins, alkaloids, tannins, sterols, flavonoids, and anthocyanins. The leaves contain alkaloids	Leaves, fruit, branch and root	Gavidia-Valencia et al. (2022), González-Velázquez et al. (2021)	Antimalarials and bactericides demonstrated *in vitro* tests
*Artemisia annua* L	Asteraceae	Castilian: Ajenjo, Ajenjo anual, ajenjo dulce, ajenjo chino Kichwa: Yura amawta, yura yachak; yura yachay; Yura yachachic	Fever, chills, hemorrhoids, anemia, fatigue, heatstroke, convulsions, pain, dental swelling, dysentery, cholera, eye problems and skin diseases	Antiparasitic, Anti-inflammatory, Immunoregulatory, Antioxidant, Biopesticide, Antimalarial Antimicrobial	Sesquiterpenoids, triterpenoids, flavonoids, coumarins, and phenols; and a smaller proportion of alkaloids, essential oils, saponins, and polysaccharides	Fruit and leaf	Chebbac et al. (2023), Czechowski et al. (2019), Gupta et al. (2009), Mohammadi et al. (2020)	*In vitro* bioactivity of artemisinin against *P. falciparum;* *In vitro* Antimicrobial, antioxidant and anti-Listeria activity
*Clinopodium nubigenum* (Kunth) Kuntze	Lamiaceae	Castilian: Sunfo, Poleo de llano, tipo de llano Kichwa: Pagalu (Annobon), Uchumarca	Infusions: (Leaves and flowers) colds, flu, stomach aches, dysentery and menstrual cramps	Insecticide, Plagicide, Antimicrobial, Antibacterial	Essential oil: carvacrol acetate, carvacrol, p-cymene, thymol, eugenol, and sesquieterpenes; pulegon, citronellyl acetate, and citronellal; 1-octen-3-yl acetate, an alcohol, 3-octanol, and a nonanal aldehyde	Leaves and flowers	Bedini et al. (2019), Noriega et al. (2018)	Antioxidant and antimicrobial activity against respiratory pathogens; antibacterial, antifungal, and *in vitro* acetylcholinesterase inhibitory activity of myiasis *Lucilia sericata*
*Jungia rugosa* Less	Asteraceae	Castilian: Carne humana, fompo, guayombo Kichwa: Tikache, matico serrano, matico de puna, matico blanco, karamati	Leaves (infusion): For baths and branches as a rope, to treat blows, inflammation of the urinary tract, gastric ulcers, healing and for coughs	Antibacterial, Anti-inflammatory, Hypoglycemic	Phenols, amino acids, flavonoids, tannins, alkaloids, anthraquinones, catechins, terpenes, saponins, carbohydrates, sesquiterpene lactones and sesquiterpene furans	Leaves	Calvopiña et al. (2021), Wilches et al. (2015)	Cholinergic activity *in vitro* and acute and chronic anti-inflammatory activity in rodents *in vivo*
*Minthostachys mollis* (Kunth) Griseb	Lamiaceae	Castilian: Menta andina, tipo, poleo, pepirina Kichwa: Muña	Stomach pain, flatulence, vomiting, rheumatic conditions, bronchitis, colds, asthma, and as a sedative. As a food preservative, insect repellent, biopesticide, and antifungal	Carminative; Analgesic; Antispasmodic; Gastroprotective	Essential oil: menthone, neomenthol, menthol, menthol, pulegone, carvacrol, thymol and D germacrene and limonene	Fruto y hojas	Huamaní et al. (2021), Juncos et al. (2024), Rojas-Armas et al. (2019)	Acute oral toxicity *in vivo* at doses of 500 mg/kg for 28 days of the essential oil in rats; Antifungal activity against *Candida albicans* *in vitro*
*Peperomia galioides* Kunth in H. B. K	Piperaceae	Castilian: Kichwa: Congona del monte, Mishki congona de la sacha, Sacha congona, Congona silvestre, Menta	The fruits are edible; culturally, the leaves are used to ward off “bad air,” in rituals against the evil eye, and in good luck baths. Medicinally, they are used for fever, nerves, postpartum baths, colic, and mumps; and the stem is used for deafness	Antibacterial; Antiparasitic; Anti-inflammatory; Sedative; Cicatrizant	Flavonoids, coumarins, phenols, triterpenes, steroids and tannins, sesquiterpenes and monoterpenes	Whole plant	Fournet et al. (1996), Langfield et al. (2004), Mahiou et al. (1996), Wilches et al. (2021)	Antiparasitic activity of prenylated quinones extracted from *P. galioides* against three Leishmania species *in vitro*; Antibacterial activity against *S. aureus* and *S. epidermidis*; Anti-inflammatory and sedative activity *in vivo* in mice
*Carica pentágona* Heilborn	Caricaceae	Castilian: Babaco Kichwa: Turunchi	Its fruit is for food consumption	Vasodilator; Anti-inflammatory; Immune system modulator; Detoxifier	Water, proteins, carbohydrates, fiber, fat and other compounds such as sodium, potassium, calcium, phosphorus, riboflavin, sulfur, carotenes, thianine, pyridoxine and ascorbic acid	Fruit	Aguirre-Rodríguez et al. (2024)	Functional activities and bioactive compounds *in vitro*

### 3.3 Approach to the population and respective validation of the platform

As mentioned in the methodology section, each monograph developed was validated using a double-blind methodology, with an initial review by the editor, subsequent assignment to the administrator, and distribution to academic peers who evaluated the information compiled for each species. As a next step, the aim was to make organized knowledge accessible to the population, following criteria for the development of knowledge transfer programs related to production, research trials, and therapeutic treatments (Caminero et al., 2021).

The first point of contact between the Herbal Pharmacopoeia of Ecuador and Ecuadorian citizens is its digital platform, which is publicly accessible at www.farmacopea.ec. The site features two main links: “Farmacopeadia,” which contains various cultivation guides, plant preparation methods, etc., and the “Farmacopea,” where all the monographs are available in Spanish, Kichwa, and soon in English. The inclusion of Kichwa is one of the strategies to strengthen this connection, offering summaries that can be downloaded for free from the digital platform, promoting the dissemination of knowledge in an accessible and culturally relevant format.

### 3.4 Integrate the outcomes from the development and implementation of the website

The development and launch of the digital platform for the Herbal Pharmacopoeia of Ecuador enabled the establishment of a modern, secure, and accessible virtual space for disseminating national herbal knowledge. The platform provides access to monographs, scientific articles, and academic services, promoting integration among member institutions of the FAPRONAT Consortium and the broader academic community. Additionally, it has expanded its reach through active social media, thereby strengthening the project’s visibility and impact at both national and international levels. This technological tool not only enhances the accessibility of specialized information but also fosters the appreciation of ancestral knowledge and promotes research in Ecuadorian phytotherapy.

## 4 Discussion

Our research shows that over 576 scientific documents were published in SCOPUS between 2000 and 2024, focusing on the keywords “Pharmacopoeia” and “Natural Products,” revealing a positive trend in scientific publications. This upward trajectory indicates that ongoing research effectively addresses existing gaps in scientific knowledge, with expectations for sustained growth in international journals. The United States and Brazil have emerged as key contributors to scientific output in the Americas, with their Pharmacopoeias playing a crucial role in ensuring the quality of medicinal products (Haiyan and Chuncai, 2022; Wang et al., 2022). The United States Pharmacopoeia has established vital global quality standards and is a reference for the pharmaceutical industry. In the Brazilian Pharmacopoeia, traditional medicine is strongly emphasized, tailoring its focus to meet the specific needs of the population. This pattern can also be observed in other internationally recognized Pharmacopoeias, such as Egypt, Europe, India, Japan, Korea, and China (Haiyan and Chuncai, 2022; Wang et al., 2022).

This global context highlights the need for the harmonization of pharmacopoeias to enhance international pharmaceutical trade and improve patient access to safe and effective treatments (Qu et al., 2018; 2022). Exploring key terms such as phytochemistry, therapeutics, chemistry, herbal medicine, and medicinal plants has underscored its significance. This central theme connects diverse research fields and paves the way for development and innovation in plant-derived pharmaceuticals, as shown in the results section (Figure 4). The Herbal Pharmacopoeia seeks to resolve issues stemming from insufficient coordination and collaboration among academic stakeholders, thereby minimizing the duplication of efforts associated with revisiting previously studied topics. Securing funding for research projects can often prove challenging, and this initiative will contribute to more efficient investments in scientific research (Gasmi et al., 2024).

In Ecuador, the sustainability of the Herbal Pharmacopoeia relies heavily on securing funding, a critical element for any organization’s growth and international relevance. In contrast, pharmacopoeias in Asia often receive substantial financial support from governments, international organizations, and the pharmaceutical industry. This backing allows them to establish high-quality standards and scientifically validate traditional medicine. Such support is vital for global recognition; without a robust Pharmacopoeia, scientific collaboration suffers, access to funding diminishes, and the ability to publish in high-impact journals is hindered. Consequently, local innovation is restricted, resulting in a greater dependence on external research (Shen et al., 2021; Gasmi et al., 2024). An official Herbal Pharmacopoeia will help regulate and certify natural medicinal products, and establish a solid foundation for local scientific research, particularly in traditional medicine and phytotherapy. It will document the usage of medicinal plants, their efficacy, and safe dosages, paving the way for the development of therapeutics that address public health needs while stimulating the economy. The absence of this official compilation limits countries’ healthcare safety and their capability to engage in the global scientific and economic spheres (Gasmi et al., 2024; Shen et al., 2021). Much like many other nations, Ecuador must invest in scientific research, the development of Pharmacopoeias, and the production of natural products. Attention should be directed toward training scientists in this field and fostering international collaboration, as exemplified by Brazil, a leader in scientific output in this arena (Table 2). Although Ecuador has made strides in natural product research, it lags behind its regional counterparts. A significant limitation is the lack of an organization that can compile and systematize scientific and traditional knowledge within the country, as well as the capacity to identify research trends genuinely relevant to Ecuador clearly (Leong et al., 2020).

The advancement of scientific production is strongly linked to investments in research. Countries investing more in this area typically benefit from superior infrastructure, equipment, and reagents, which enhance scientific output. Additionally, the presence of qualified personnel and the fostering of international collaboration among researchers are vital factors that contribute to this growth, as supported by various studies (Cuéllar Rodríguez, 2023; Guzmán-Cervantes et al., 2024). The future perspectives of the Herbal Pharmacopoeia in Ecuador are promising, especially considering its rich biodiversity and ancestral knowledge about health, which strengthen complementary medicine and the country’s economy. Studies indicate that systematizing herbal knowledge in an official pharmacopoeia promotes research development to ensure the quality and efficacy of phytotherapeutic resources (Rastogi et al., 2022a). This effort contributes to integrating traditional medicine into the public health system and helps regulate its safe use, in line with WHO recommendations (Burton et al., 2014; WHO, 2024b). Additionally, the WHO emphasizes the importance of these practices in countries with high biological diversity, as a national pharmacopoeia supports scientific research on bioactive compounds that can offer low-cost, highly effective therapeutic solutions for local and emerging diseases (Mosquera, 2022).

The Herbal Pharmacopoeia and its promotion of research on medicinal plants will stimulate studies on phytochemicals in Ecuador, thereby expanding pharmacognosy and validating traditional knowledge (Gonzalez et al., 2022). This aspect is particularly relevant in the context of antimicrobial resistance, as the herbal pharmacopoeia will support the exploration of natural sources of antimicrobials, which are crucial in addressing current challenges, such as multidrug-resistant “superbugs” (Gharebaghi et al., 2020). Additionally, research indicates that compounds derived from Ecuadorian plants have potential for treating chronic diseases, highlighting the relevance of a digital research platform that documents and validates their therapeutic effects (Valencia, 2020).

The existence of an official pharmacopoeia impacts the regulation of medicines and health products, fostering solid and continuous scientific production. In countries such as China, the United States, Germany, and Japan, these pharmacopoeias set standards for research, development, and drug evaluation, ensuring data are replicable and comparable internationally, encouraging innovation, and improving medical treatments. They also foster collaboration among universities, research centers, and the pharmaceutical industry, resulting in high-impact publications and the development of new therapies (Rastogi et al., 2022b; The International Pharmacopoeia, 2013). In contrast, in much of Africa, Latin America, and Southeast Asia, the absence of a uniform regulatory framework limits research, the validation of plant-based products, and the utilization of traditional knowledge, thereby hindering development and export potential (Yapar and Mehmet, 2020).

The network representation demonstrates how national, regional, and international pharmacopoeias are interconnected, forming a framework for collaboration and regulatory harmonization. As shown in the WHO Index of Pharmacopoeias, the existence of multiple pharmacopoeial authorities reflects the diversity of regulatory approaches, while also highlighting the need to establish links that enable the convergence of standards for the quality, safety, and efficacy of medicines. In Figure 3, the centrality of pharmacopoeias, such as those of the United States, China, India, and the European Pharmacopoeia, highlights their role as global regulatory references. Meanwhile, regional connections, including the Eurasian Economic Union and the African initiative, demonstrate collective efforts to strengthen technical cooperation. This interrelationship suggests that progress toward international harmonization depends not only on the production of regulatory texts but also on the construction of strong networks of communication and scientific consensus (WHO, 2023). The *Herbal Pharmacopoeia of Ecuador* is expected to have a positive impact on the rural economy and environmental conservation. Studies indicate that the sustainable management of medicinal plants in Ecuador offers economic alternatives for indigenous and rural communities, while also promoting ecosystem preservation (Robles Arias et al., 2020; Tinitana et al., 2016). These efforts demonstrate a commitment to sustainable development, promoting the safe and regulated use of natural resources while strengthening the health and socioeconomic sectors through policies that support the use and conservation of biodiversity (Burton et al., 2014; Mosquera, 2022). Ecuador faces the challenge of preserving and scientifically validating the use of medicinal plants. Although numerous articles highlight their medicinal importance, most lack support from phytochemical, pharmacological, or toxicological evaluations, which hinders their integration into formal health systems. In response to this situation, the WHO, in its Draft Global Traditional Medicine Strategy (2025–2034), emphasizes the need to incorporate herbal medicine into alternative therapies based on scientific criteria, ensuring their quality and reducing the risk of toxicity for the population (WHO, 2024a; World Health Organization, 2024). Ensuring the effectiveness of the herbal pharmacopoeia in Ecuador requires documenting, standardizing, and protecting both scientific and ancestral knowledge, as well as establishing parameters that certify the quality and safety of phytomedicines. Pharmacopoeias play a key role in regulating herbal medicine, enabling its integration into the formal healthcare system. However, their consolidation depends on the generation of reliable evidence and production standards that guarantee the uniformity and safety of the products (Rastogi et al., 2022a).

The FAPRONAT Consortium has emerged to gather scientifically validated information and preserve valuable ancestral knowledge by safeguarding it in an Herbal Pharmacopoeia. One of the collateral outcomes of this work is the development of knowledge transfer programs related to production, the development of trials, and therapeutic treatments (Caminero et al., 2021).

The first interaction between the Herbal Pharmacopoeia of Ecuador and the public is through its digital platform, which is freely accessible. It contains two main sections: “Farmacopeadia,” which includes various guides on cultivation, methods of plant preparation, and more; and the “Pharmacopoeia” section, where all the monographs are available. Including Kichwa is one strategy that strengthens this connection with the Ecuadorian population. Summaries can be downloaded from the digital platform free of charge.

## 5 Conclusion

The analysis conducted with VOSviewer reveals that herbal pharmacopoeia significantly contributes to a country’s scientific output by collecting, organizing, and documenting knowledge about medicinal plants. It serves as a key reference for regulation and quality in the manufacturing of natural products in Ecuador, as is the case in countries such as China, India, the USA, and Brazil. In the case of Ecuador, the official establishment of an Herbal Pharmacopoeia will allow for the systematization and validation of its vast wealth of traditional and ancestral medicine, thereby strengthening scientific development and phytosanitary regulation. This progress will facilitate the analysis of bioactive compounds from native plants with therapeutic potential for chronic and infectious diseases. It will promote an evidence-based approach to medical care that enhances the safety and efficacy of natural products.

In addition, this initiative could address global challenges, such as antimicrobial resistance, by exploring plant-derived compounds for new antimicrobials while also preserving cultural and biological heritage. The inclusion of Indigenous communities in this process will ensure knowledge transfer, support the protection of ancestral wisdom, promote sustainable practices, and position Ecuador as a leader in ethnobotanical research and the responsible use of its megadiversity. Consolidating the herbal pharmacopoeia in Ecuador requires joint work among the State, academia, industry, and non-governmental organizations. Cooperation among these sectors is key to scientifically validating medicinal plants and ensuring their quality. Harmonizing criteria and maintaining production standards will be difficult without sustained support and clear regulations. Only through an inter-institutional collaboration model will it be possible to strengthen its development and ensure tangible benefits for the population.

In summary, the Ecuadorian Herbal Pharmacopoeia is a dynamic and evolving project that aims to consolidate scientific, traditional, and ancestral knowledge of medicinal plants through the use of digital technologies. The annual and continuous incorporation of new monographs is planned, together with the periodic review and updating of existing ones, to ensure their validity and scientific rigor. Additionally, the publication of an English version of the monographs is being considered, which would significantly enhance their international dissemination. The FAPRONAT consortium also remains open to establishing further collaborations and is evaluating the implementation of clinical studies to validate the efficacy and safety of Ecuador’s herbal resources.

## Data Availability

The original contributions presented in the study are included in the article/supplementary material, further inquiries can be directed to the corresponding authors.
